# Schizophrenia and Hyperostosis Frontalis Interna with History of Head Injury

**DOI:** 10.1155/2021/6634640

**Published:** 2021-08-12

**Authors:** Fatima Elghazouani

**Affiliations:** Department of Psychiatry, Materno-Infant and Mental Health Research Laboratory, Faculty of Medicine and Pharmacy, University Mohammed The First, Oujda, Morocco

## Abstract

Hyperostosis frontalis interna is an irregular thickening of the frontal bone. Its etiology is unknown. It has been rarely linked with schizophrenia and head injury. *Case Presentation*. We describe an unusual case of a 44-year-old female with schizophrenia and hyperostosis frontalis interna having a history of head trauma. At the age of 3 years, she had a head injury that could be classified as mild traumatic brain injury. She presents a family history of schizophrenia. She was admitted for resistant schizophrenic disorder. The cranial computed tomography showed bilateral and asymmetrical hyperostosis of the frontal bone that was more pronounced on the right side. This corresponds to the impact of the trauma with frontal atrophy without any metabolic or endocrinal abnormalities. *Conclusion*. We surmise that the long-term pathological effects of traumatic brain injury, including hyperostosis frontalis interna, are likely to interact with genetic vulnerability and may lead to schizophrenic disorder.

## 1. Introduction

Hyperostosis frontalis interna (HFI) is an irregular thickening of the frontal bone that is generally presented as single or multiple bilateral nodules on the inner lamina, while diploe and external lamina of the bone remain unaffected. Its mode of formation and growth is unknown. It has been linked with many symptoms. Two syndromes have been described in association with it. Morgagni's syndrome consists of HFI and both obesity and hirsutism and the Stewart-Morel syndrome comprises neuropsychiatric symptoms and headache.

Here, we describe an unusual and interesting case of schizophrenia and hyperostosis frontalis interna (HFI) having a history of head injury without metabolic or endocrine dysfunctions.

## 2. Case Report

In this report, we describe an unusual case of a 44-year- old female with hyperostosis of the frontal bone. The patient is a single Moroccan female with a university degree and living with her parents. She showed normal psychomotor development during early childhood.

**Figure 1:**
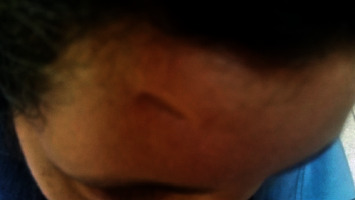
Scar of head injury.

At the age of 3 years, she had a head injury. She fell on a sharp object that caused frontal head impact (Figure 1). The bleeding was stopped at home with traditional first aid. She was somnolent on the day of trauma.

Prior to this incident, she had never been examined nor had she undergone any neurological tests, despite the persistence of moderate headaches.

She was a sociable child and had no issues in her adolescence. Family history revealed that her sister was diagnosed with schizophrenia.

The schizophrenic symptoms of our patient began when she was 22 years old. She was hospitalized and received antipsychotic treatment.

The evolution was marked by very short remissions and numerous relapses due to treatment discontinuation.

With time, the symptoms appeared resistant and severe. She had major anxiety, depersonalization, derealization, and obsessive symptoms. She presented audiovisual hallucinations with mental automatism, persecution delusion, reasoning difficulties, impaired judgment, sexual disinhibition, and emotional impulses. These were associated with several suicide attempts that did not involve sadness in her mood.

Psychometric evaluations revealed scores of 102 on the Positive and Negative Syndrome Scale (PANSS) for schizophrenia (Table 1) and 9/10 on the clock drawing test (CDT) [1, 2]. A neurological examination showed normal findings.

**Figure 2:**
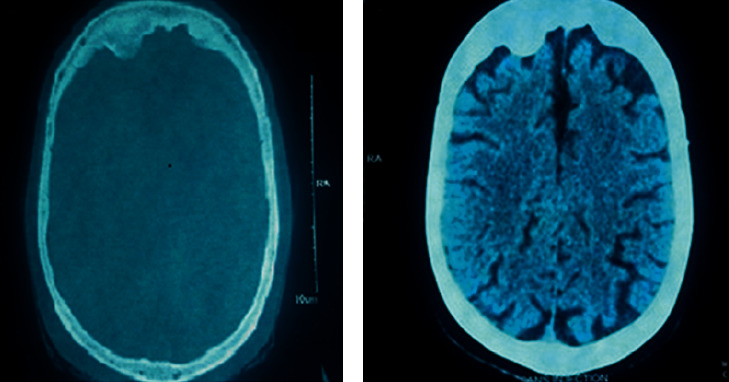
(a) Axial cranial computed tomography scan illustrating the bilateral and asymmetrical thickening of the inner table of the frontal bone. (b) Axial cranial computed tomography scan illustrating the frontal atrophy.

In the context of therapeutic resistance, cranial computed tomography was carried out. The results showed bilateral and asymmetrical hyperostosis of the frontal bone that was more pronounced on the right side. This corresponds to the impact of the trauma with frontal atrophy (Figure 2).

Although she was an obese and premenopausal woman, she showed no signs of hirsutism. Laboratory tests did not show metabolic or endocrinal abnormalities like imbalanced sexual hormones and thyroid function. She had no history of epilepsy and the electroencephalography did not show any abnormal activity.

We could not carry out further treatments, including administering clozapine and electroconvulsive therapy. During a 10-day treatment with clozapine, she developed severe diarrhea without fever or any infective cause. Moreover, her family refused electroconvulsive therapy.

## 3. Discussion

HFI has been identified in antiquity and has increased during the last century, especially among young individuals. It has been shown to be more common and more severe in females. It is usually an incidental finding on imaging, for example, in X-ray, cranial computed tomography, or magnetic resonance imaging.

It is considered an independent condition that is benign and incidental, although in some reports it has been associated with many conditions, including metabolic and endocrine disorders, headaches, epilepsy, cognitive impairment, and psychiatric or behavioral disorders. The etiology of HFI remains unknown, but it is generally related to an endocrine imbalance, especially in pituitary function. However, to our knowledge, only one report attributed HFI to head trauma [3].

Our patient presented with an unusual association of resistant psychotic symptoms, head injury, family history of schizophrenia, and HFI. Therefore, we aimed to assess whether there is a relationship between schizophrenia, head injury, and HFI.

We assume that the head injury of our patient might be one of the reported risk factors of HFI, especially because this is more developed on the side of the trauma. Moreover, our patient did not present any metabolic or endocrinal abnormalities. The head injury in the other reported case was more severe than in our case [3].

The literature has reported traumatic brain injury (TBI) as a source of neuroendocrine disturbances. Patients can develop hypopituitarism in the postacute phase of a TBI and it may normalize later; however, it may recur after the postacute phase [4]. This suggests that TBI could contribute to the formation of HFI through hypopituitarism. However, in our case, we do not have enough evidence to confirm that the traumatic event can be defined as TBI. Children may be unable to report some symptoms immediately after traumatic events, such as sensory problems, confusion, or headaches. Somnolence could indicate cerebral damage, while headache is a common and persistent symptom following mild TBI. The trauma in our case could be classified as mild TBI.

Several papers have noted an association between severity of HFI and the cortical atrophy with severity of psychotic symptoms [5]. Some authors have observed that prognosis did not seem to be affected by HFI, and the clinical outcome was in accordance with the psychiatric diagnosis.

The problem regarding our patient lies is in distinguishing whether the onset of schizophrenic disorder was a consequence of HFI; is related to childhood TBI; or that the co-occurrence of schizophrenic disorders, head injury in childhood and later development of HFI is just a coincidence, especially that the available literature has suggested that there is an increased risk of schizophrenia following TBI, with a larger effect in individuals from families with an elevated genetic predisposition to psychosis. Furthermore, some studies have supported that childhood trauma may have a causative role in some presentations of psychosis. The explanation for this long latency from injury to onset of illness may be related to the neurodevelopmental theory of schizophrenia.

Head injury, HFI, and schizophrenia are important factors that must be considered when interpreting frontal atrophy in the case of our patient. The longest follow-up TBI studies have supported that there is a brain volume loss in a patient with chronic brain injury [6]. The neuroinﬂammatory reactions following TBI may play a role in the progression of degenerative brain changes for months to years after injury [6].

Similarly, May et al. noted that the presence of HFI suggests a decrease in brain volume and has major clinical signiﬁcance as it may indicate the beginning of degenerative processes of the brain [7]. However, in our case, it is difficult to ascertain the direction of this relationship because the patient underwent a scan only once, without being examined by nuclear magnetic resonance imaging, which limited the possibility of drawing conclusions about causal relationships.

On the other hand, patients with untreated schizophrenia lasting 5 to 47 years had less cortical thickness relative to healthy people [8].

## 4. Conclusions

Finally, we suggest that all these factors (head injury, HFI, and schizophrenia) contribute to cerebral atrophy, severity, and resistant psychotic symptoms in our case.

This observation indicates that even mild head injuries have the potential to initiate a cascade of neuropathological events that reduce brain reserve capacity and increase vulnerability for neuropsychiatric disorders.

In conclusion, we surmise that the long-term pathological effects of TBI including HFI may represent an additional causal factor that can interact with genetic vulnerability and may lead to schizophrenic disorder.

## Figures and Tables

**Table 1:** Patient's PANSS subscale score.

	Score/maximum score
PANSS positive subscale score	31/49
PANSS negative subscale score	20/49
PANSS general psychopathology subscale score	51/112
PANSS total score	102/210

PANSS: Positive and Negative Syndrome Scale.

## Data Availability

No data were used to support this study.

## Abbreviations

HFI: Hyperostosis frontalis interna
TBI: Traumatic brain injury
PANSS: Positive and Negative Syndrome Scale
CDT: Clock drawing test.
